# An improvement on the assessment of ecosystem services value of urban wetlands under consideration of water yield at regional scale

**DOI:** 10.1371/journal.pone.0306628

**Published:** 2024-09-09

**Authors:** Lijun Wu, Zui Hu, Fuwei Huang

**Affiliations:** 1 College of Geography and Tourism, Heng Yang Normal University, Hengyang, China; 2 National and Local Joint Engineering Laboratories for Digital Protection and Creative Utilization of Traditional Village and Town Culture, Hengyang, China; 3 Institute of Education and Examination, Education Bureau of Hunan Province, Changsha, China; Chengdu University of Information Technology, CHINA

## Abstract

Urban wetlands are gaining more attention and showing more important play in the sustainability. Surge findings are attached on the assessment of Wetland Ecosystem Service Value (WESV) in urban areas. While determining WESV in urban areas, it is still difficult to capture the nature of wetlands due to neglecting the impact of related impervious surfaces. It is necessary to improve the existing evaluating methods of WESV when seeking the truth. In order to narrow this issue, based on InVEST model, this study employed Equivalent Factors (EF) to determine WESV in urban areas with a case of Hengyang City, China. The main materials of this study included high-resolution images, DEM, precipitation, evapotranspiration, soil, vegetation, and statistical yearbook of the case. By comparing the uncorrected results with precipitation corrected and water yield corrected results of WESV, this study confirmed that: (1) the corrected results can reflect more real status than uncorrected; (2) in terms of EF, the water yield factor is more conducive to finding the truth than precipitation. Through this study, the water yield factor can effectively reduce the adverse effects of climate and improve the accuracy when determining WESV in urban areas.

## Section 1: Introduction

Urban wetlands, as an important component of urban ecosystems, play active roles in biodiversity, water conservation and climate changes [1, 2]. Urban wetlands are also of enormous significance to bolstering the development of social economy, such as tourism, scientific research, and public education [3]. However, there are still certain unreasonable activities or behaviors that may damage or degrade the ecological functions of urban wetlands such as filling the lakes in urban areas [4]. These may bring some irreversible damage to the sustainable development of cities [5]. Therefore, much more attention and efforts are of great sense for the preservation and further understanding of crucial functions of urban wetlands [6]. This raises an urgent need to assess the Wetland Ecosystem Services Value (WESV) in urban areas to grasp the nature of urban wetlands.

Over the past decades, rapid urbanization has had a profound impact on China and the world [7, 8] and most cities continuously experienced fast sprawls. This enormously impacted the urban wetlands of cities. A great number of archives focused on this, such as potential water crises, air pollution, biodiversity loss, and climate warming [9]. Although rich findings concern the assessment of WESV in urban areas, there is still a few issues that need to be refined. For instance, many researchers put their interests on the entire features of WESV of a single city or urban agglomeration in a large region [10, 11]. These works usually neglected the slight differences in various surfaces or nuances of ecosystem services of urban wetlands within each city. So they cannot adequately reflect the spatial heterogeneity within the urban environment. This makes it difficult to catch the real status of WESV in urban areas. In order to seek the truth way for examining the impact of human activities, it is significantly to recognize the differences of WESV between urban built-up and non-built-up areas. In fact, the outstanding differences in ecosystem services functions between urban built-up areas and non-built-up areas [12] can be easily investigated.

In terms of the Millennium Ecosystem Assessment (MEA) [13], which was launched in 2005, the functions of ecosystem services mainly include four categories, regulating services, provisioning services, supporting services, and cultural services, respectively [14]. In essence, many scholars strove to deeply understand the ecosystem services through MEA. For example, Xie G.D. et al. developed an integrated method to dynamically determine ESV by the Value Equivalent Factor (VEF) in the unit area [15] and widened the range and scope of ecosystem services according to 4 types of regional ecosystems. From the current documents, the mainstream approaches to evaluating ESV [16–18] mainly included the Functional Value Method (MVF) and VEF. Estimating the value of wetland ecosystem services can provide a basis for restoring wetland functions [19]. By employing the energy value method and the logarithmic average partition index decomposition method to calculate the primary ecosystem service values of wetlands, and analyzing the contributions of different driving factors to the variations in ESV, strategic insights are provided for wetland conservation [20, 21]. Besides, certain scholars thought that the wetland ecosystem services could also be divided into the final services and the intermediate services. For example, Cui LJ (2016) employed various ecological economic methods to calculate ESV [22] from this perspective. At the same time, many mathematical equations or models had also been introduced to determine or predict the development trend of WESV, such as the structural equation model [23], and the system dynamic model [24], etc.

However, there is still an enormous knowledge gap to improve the calculation results of WESV under various conditions. This attracts many scholars to contribute their models or methods. Noted that, VEF is one of the most popular methods to correct the calculation results of ESV due to its small data volume and clear types of ecosystem services functions. For example, Xie G.D. et al. [15] developed a spatiotemporal dynamic adjustment model by introducing a couple of factors, such as Net Primary Productivity (NPP) of vegetables, precipitations, soil retention, etc. [15, 25, 26]. More influence factors are involved in improving the assessment results of WESV, such as social willingness to the payment, land use types, and areas [27, 28]. It is important to point out that although numerous works and research have focused on the improvement of ESV, the research specialized in improving the assessment of WESV is very rare and deficient. In order to explore this issue, the work tried to integrate the water yield factor and spatiotemporal regulation factor of precipitation to precisely determine the WESV in urban areas. Because the water yield factor links the precipitation, evapotranspiration, soil, land uses, and Plant Available Water Content (PAWC) [29] together to determine the amount of water production per unit area at a regional scale. Compared to precipitation, the water yield factor can comprehensively reflect more environmental features.

The main objective of this work was set as seeking a feasible way to accurately evaluate WESV in urban areas through employing water yield factor to correct the apparent errors when concerning VEF. In addition, the improvement of calculation ideas or methods on ESV would also benefit from the case of this paper. To examine the effects of improvements in WESV assessment of urban wetlands while considering the water yield factor, this work selected Hengyang City (located in Hunan Province, China) as the case and utilizes high-resolution remote sensing images to determine WESV. The remainder of this work was structured as follows. Section 2 briefed the study area and details the materials. Section 3 showed the methods. Section 4 analyzed the results. Section 5 drew the conclusions in this work.

## Section 2: Materials and methods

### Study area

**Fig 1:**
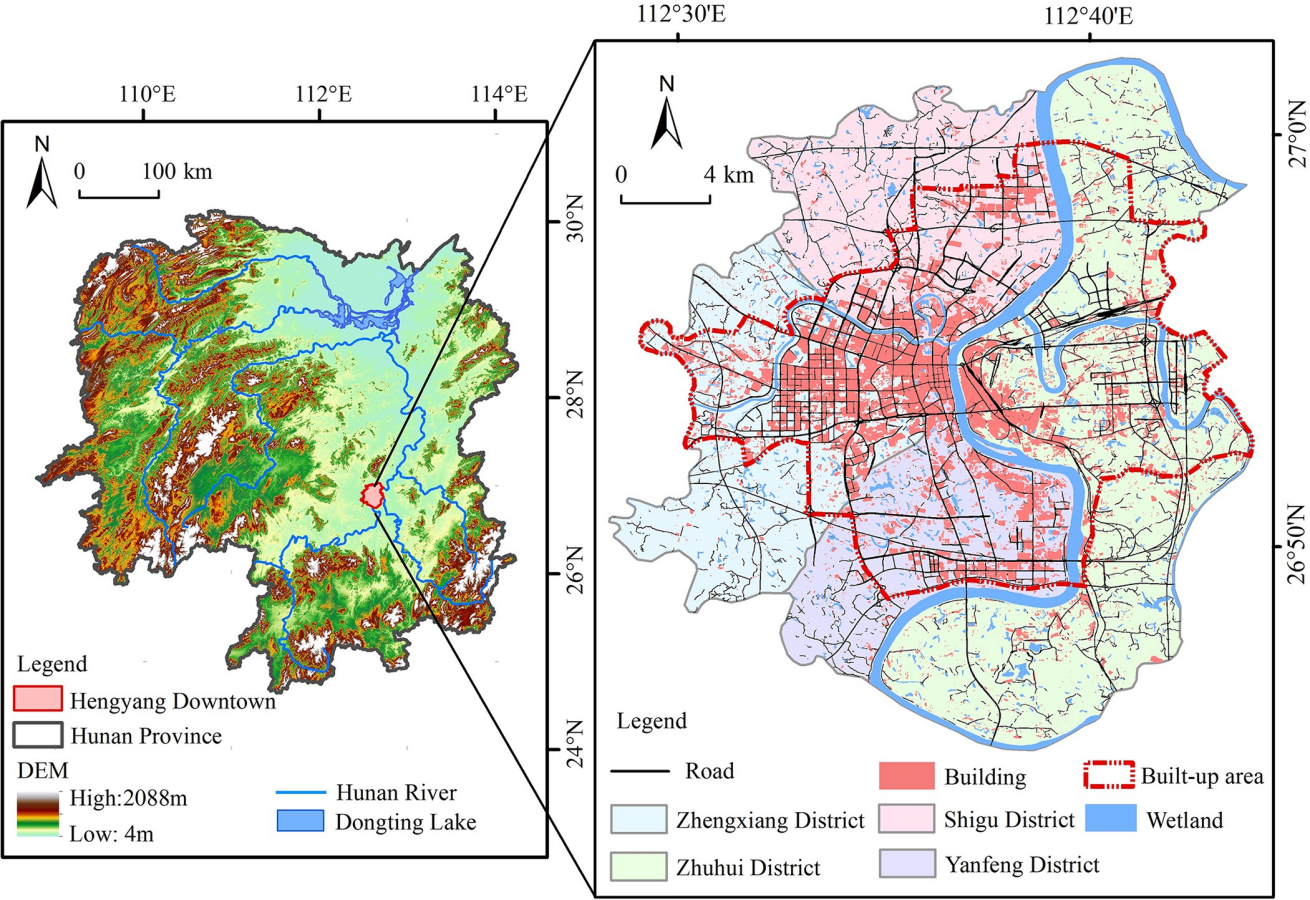
Distribution map of the study area. (PS: The drawing number is GS (2020) 4619, and the base map is not modified). Republished from Acta Ecological Sinica under a CC BY license, with permission from Shujin He, original copyright 2018.

Hengyang City is situated in southern China and the central-southern areas of Hunan Province (108°47′~ 114°15′E, 24°39′~ 30°08′N) (Fig 1) and simultaneously located in the middle reaches of Xiangjiang River (Which is the longest river of Hunan Province and is also a main tributary of the Yangtze River in the southern areas of China). Hengyang City extends across the regions with latitude ranging from 26°45′5″N to 27°01′19″N and longitude covering from 112°29′7″E to 112°43′55″E (Fig 1). The relief of Hengyang City is higher in the south and lower in the north. The climate of Hengyang is characterized by a typical subtropical monsoon climate, with hot and humid summers, mild and dry winters, and significant temperature variations in spring and autumn. The abundant rainfall supports the growth of crops and the development of the urban ecosystem. In Hengyang City, the average annual temperature is 18°C and the annual precipitation is 1500 mm.

The urban areas of Hengyang City are composed of 5 administrative districts, including Zhuhui District, Yanfeng District, Zhengxiang District, Shigu District, and Nanyue District, respectively. However, Nanyue District is far away from the main urban areas and is separated by Hengshan County of Hengyang City. The trunk of the Xiangjiang River runs through the main urban areas of Hengyang City from south to north. And, the other two important tributaries of the Xiangjiang River merge into the trunk in the main urban areas of Hengyang City, one is the Zhengshui River, the other is the Leishui River. This forms a very typical geographical landscape ‘three-shuikou-lock-river trunk’ [30]. Hence, Hengyang City is very rich in wetlands, especially in urban wetlands. In this work, the urban wetlands in the main urban areas of Hengyang City were the sole focus, and it was noted that Nanyue District was not encompassed.

The total area of the four administrative districts in the main urban areas of Hengyang City (Zhuhui District, Yanfeng District, Zhengxiang District, and Shigu District) amounts to 51, 830.58ha This accounts for 3.39% of the entire land area of Hengyang City. According to the statistical yearbook, to the end of 2021, the Gross Domestic Production (GDP) of the former 4 districts is about 1,051.9 × 10^8^ yuan, the volume of the whole population is 1, 352, 100, and the entire grain farming area is 4,540.43 ha, and the total value of grain products is about 86 × 10^8^ yuan [31].

### Materials

In order to precisely observe the urban wetlands of the main urban areas of Hengyang City and deeply understand their natural features, high-resolution remote sensing images were employed in this work. The 5m-resolution image data were collected through Planet Satellite and the Google Earth Platform. To facilitate the comparison with features from various years, images of 2017, 2019, and 2021 were selected. Prior to the experiment launch, the entire set of image data underwent a series of preprocessing steps, including atmospheric correction, spatial correction, stitching, and cropping. According to the National Specification ‘Land Use Status Classification (GB/T 21010-2017), the land use database of urban wetlands of the main urban areas of Hengyang City was established with the help of AI Earth Platform (https://account.aliyun.com/). All of the data sources which were utilized in this work are listed in Table 1. The final results of land use classification in this work are shown in Table 1. Through Table 1, there are 7 types of land uses in the main urban area of Hengyang City: forest, grassland, farmland, water, buildings, road, and bare lands, respectively.

**Table 1:** Data source.

Data type	Data name	Data source	Applications
**Observation data**	30m resolution DEM (elevation)	NASA Earth Observatory (https://earthobservatory.nasa.gov/) (https://nasadaacs.eos.nasa.gov/)	Extraction Basins
	Precipitation data (2015-2021) (mm)	China Meteorological Science Data Sharing Service (https://data.cma.cn/)	Obtaining annual precipitation
**Experimental data**	Potential evapotranspiration dataset (2017-2020) (mm)	National Scientific Data Centre for the Tibetan Plateau (http://data.tpdc.ac.cn/zh-hans/)	Calculation of water yield
	Data on soil types and soil texture in China	Geographic Remote Sensing Ecology Network (www.gisrs.cn/)	
	Soil depth (mm)	China Soil Database of Scientific Data Center of CAS (http://vdb3.soil.csdb.cn/)	
	Evapotranspiration coefficient	Refer to UN FAO evapotranspiration factor (https://www.researchgate.net/publication/345417517_FAO_Irrigation_and_Drainage_Paper_No_56)	
	Depth to root restricting layer (mm)	Refer to the InVEST model root depth table	
	Seasonal constant	Refer to rainfall characteristics parameters	
	Watershed	Hydrological analysis of DEM data using ArcGIS to extract basins and sub-basins	
**Statistical data**	Statistical Bulletin of National Economic and Social Development	Hengyang Municipal People’s Government (http://www.hengyang.gov.cn/)	Access to grain sown area and output value

It is crucial to emphasize that the boundaries of the built-up areas in the main urban regions of Hengyang City are determined by utilizing the distribution of building lands and road lands to prevent the fragmentation of built-up area boundaries [32]. Furthermore, the minimum areas of urban wetlands in the main urban regions of Hengyang City are also determined as 1ha. Because according to the findings of Cui L.J. et al. [32], a wetland can maintain the diversity of reptiles and amphibians if its area is more than 1 ha. This is helpful to survey the various functions and features of urban wetlands of ecosystem services at the city level.

**Fig 2:**
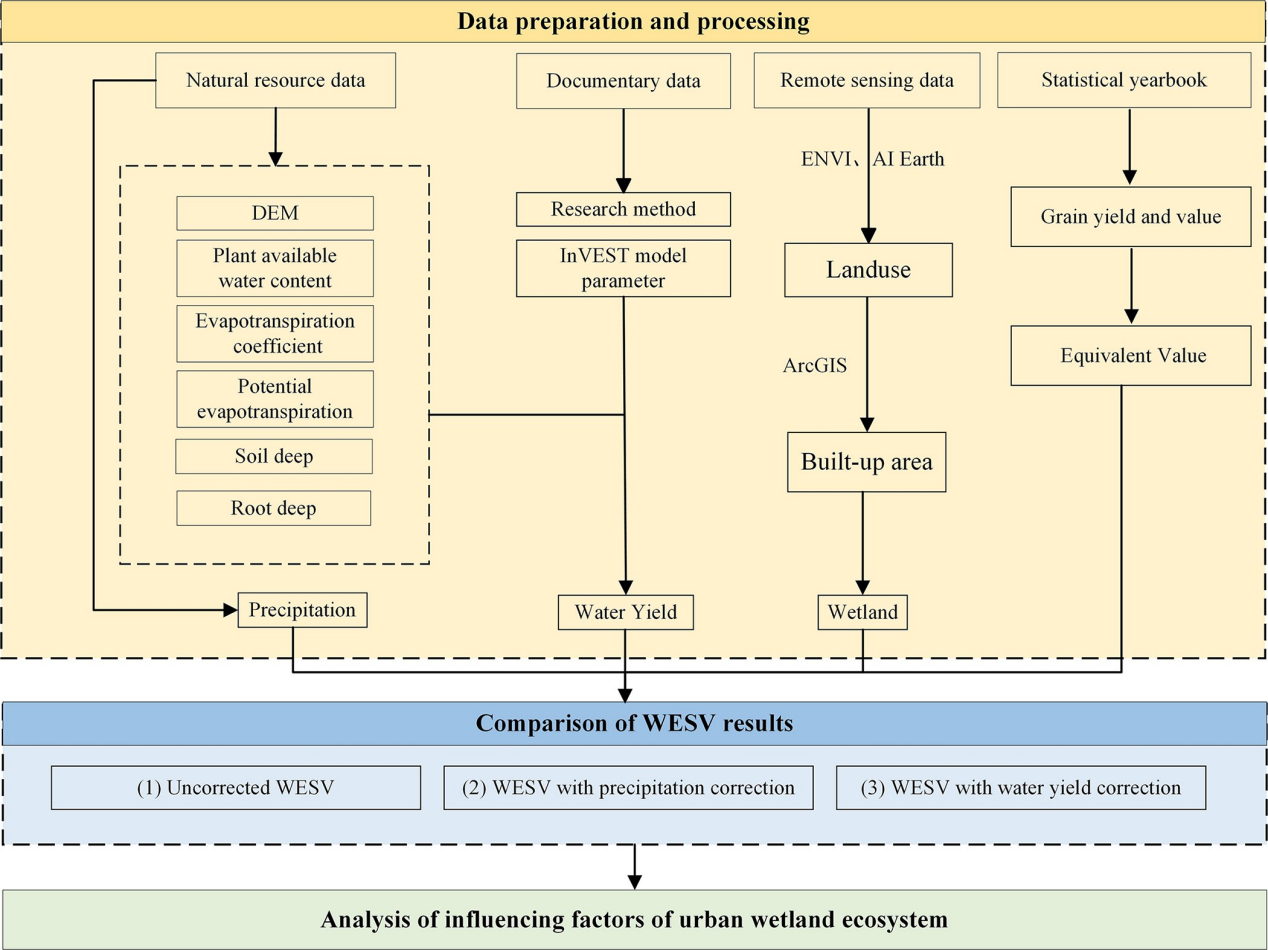
Research flow chart.

In this work, the InVEST model was employed to determine the water yield factor, and the corresponding water yield parameters were referenced in the literature [33]. Noted that, the water yield of a region is affected by the annual precipitation, land uses, and evapotranspiration; besides, the changes in climatic conditions also impact the ground runoffs [34, 35]. All of the data preparation processes and steps are outlined in diagram 2. In Fig 2, (1) Uncorrected WESV refers to the value of ecosystem services in their natural state, without undergoing any adjustments or modifications. (2) WESV with precipitation correction refers to take into account the impact of precipitation on wetlands and making adjustments for hydrological regulation and water resource supply. (3) WESV with water yield correction refers to consider with various factors beyond precipitation that influence wetlands, such as land use, soil layer characteristics, and vegetation, adjustments are made to the WESV.

## Section 3: Methods

### Water yield factor

In essence, the water yield factor is the difference between precipitations and evapotranspiration [36]. In order works, the precipitations and evapotranspiration can act as the main indicators to measure the spatial patterns of water production in a region. InVEST model covers multiple indicators including climates, soil, topography, and watersheds. InVEST model uses the water-heat coupling balance and the annual average precipitations to determine the annual water production in a region [37].

The formula for calculating the water yield factor is showed as follows (Formula 1):

$$Y(x)=\left(1-\frac{AET(x)}{P(x)}\right)∙P(x)$$ (1)

Here, *Y(x)* represents the annual water yield of each grid unit *x* in the study area; *AET(x)* represents the annual actual evapotranspiration of each grid unit *x*; *P(x)* represents the annual precipitation of each grid unit *x*. The plant evapotranspiration $\frac{AET(x)}{P(x)}$ of each land use type is determined through the Budyko water-heat coupling equilibrium hypothesis (Formula 2), which is proposed by Zhang [38].


$$\frac{AET(x)}{P(x)}=1+\frac{PET(x)}{P(x)}-{\left[1+{\left(\frac{PET(x)}{P(x)}\right)}^{\omega }\right]}^{1/\omega }$$ (2)

Here, *PET(x)* represents the potential evapotranspiration; *ω* is an empirical parameter that represents non-physical parameters of the natural climate and soil properties. Donohue et al. [39] developed a model (Formula 3) to determine *ω(x)*.


$$\omega (x)=Z\frac{AWC(x)}{P(x)}+1.25$$ (3)

In Formula 3, the coefficient Z means a seasonal constant. According to Yin G et al. (2020) [40], the calculation results of InVEST will be optimal if Z is set to 15. So does this work. In addition, *AWC(x)* represents the soil available water capacity (mm) and is calculated through Formula 4.


$$AWC(x)=Min({MaxSoilDepth}_{x},{RootDepth}_{x})*{PAWC}_{x}$$ (4)

Here, *MaxSoilDepth*_*x*_ represents the maximum soil layer depth; *RootDepth*_*x*_ represents the depth of plants roots; *PAWC*_*x*_ represents the plant-available water content, which is the differences between the field water holding capacity and the wilting point. *PAWC*_*x*_ is mainly used to evaluate the total water stored and released by the soil for plants. *PAWC*_*x*_ can be determined through Formula 5, which is proposed by Zhou et al. [41].


$$PAWC=54.509-0.132∙sand-0.003{∙(sand)}^{2}-0.055∙{silt-0.006∙(silt)}^{2}-0.738∙clay+0.007∙{\left(clay\right)}^{2}-2.688∙OM+0.501∙{\left(OM\right)}^{2}$$ (5)

Here, *sand* represents the percentage of sand particles in the soil; *silt* represents the percentage of silt particles; *clay* represents the percentage of clay particles; *OM* represents the percentage of soil organic matter.

### WESV

The equivalent factor is usually used to calculate ESV. According to the National Ecosystem Service Standard Value Equivalent Factor Table, for a region, the value of an equivalent factor is equal to one-seventh of the annual average yield value of main food crops [42]. Through Formula 6, this work calculates the economic values of a single standard equivalent factor according to the market values of rice per unit area [15].


$$P_{a}=\frac{1}{7}\times \frac{P_{w}}{S_{w}}$$ (6)

Here, *P*_*a*_ represents the economic values of the equivalent factor of WESV (yuan per hectare); *P*_*w*_ means the economic values of the output of wetland ecosystem (yuan); *S*_*w*_ is the areas of the wetland ecosystem (hectares).

In fact, for ecosystems, temporal and spatial differences can often lead to various variations of the inner structures or exterior forms. This usually brings the changes of ecological services functions and the corresponding economic values of ecosystems. In order to investigate these features, Xie G.D. et al. [15] tried to correct ESV by using temporospatial adjustment factor. In the study, Xie G.D. et al. employed precipitation (Formula 7) to carry out the correction.


$$\left\{ \begin{array}{c} R_{ij}=\frac{W_{ij}}{\bar{W}}\\ F_{ij}=R_{ij}\times F_{t} \end{array}\right.$$ (7)

Here, *R* represents the temporal-spatial regulation factor of precipitation; *W*_*ij*_ refers to the average precipitation (mm/ha) of unit area in the region *j* of year *i* in the ecosystem; $\bar{W}$ refers to the annual average rainfall (mm/ha) of unit area in the study area; *F*_*ij*_ represents the value equivalent factor of WESV in year *i* and region *j*; *F*_*t*_ represents the value equivalent factor of water resource supply or the hydrological regulation functions of ecosystem services which need to be corrected.

In order to reduce the impact on the assessment results of WESV, which are caused by the annual variations of regional rainfall, this work represents a new water yield regulation factor *T*_*ij*_ (Formula 8) by referencing the principles of precipitation regulation factor.


$$\left\{ \begin{array}{c} T_{ij}=\frac{Y_{ij}}{\bar{Y}}\\ F_{ij}=T_{ij}\times F_{t} \end{array}\right.$$ (8)

Here, *T* represents the water yield regulation factor; *Y*_*ij*_ refers to the average water yield (mm/ha) of the unit area in the area *j* of year *i*. $\bar{Y}$ refers to the annual average water yield per unit area (mm/ha).

In this work, we employ Formula 9 to determine the value of ecosystem services function per unit area and Formula 10 to calculate WESV by referencing the principles and methods of ESV [43].


$$P_{b}=F_{ij}\times P_{a}$$ (9)

Here, *P*_*b*_ means the value of ecosystem services function of urban wetlands per unit area; *F*_*ij*_ represents the value equivalent factor of water yield in the *i* year and the *j* area.

Finally, in this work, the results of WESV in the study areas are determined through the following formula.


$$ESV=P_{b}\times S_{w}$$ (10)

Here, *ESV* is the total value (yuan) of ecosystem services of urban wetlands; *S*_*w*_ is the area (ha) of urban wetlands.

## Section 4: Results and analysis

### Variations of urban wetlands in the main urban areas of Hengyang City

**Fig 3:**
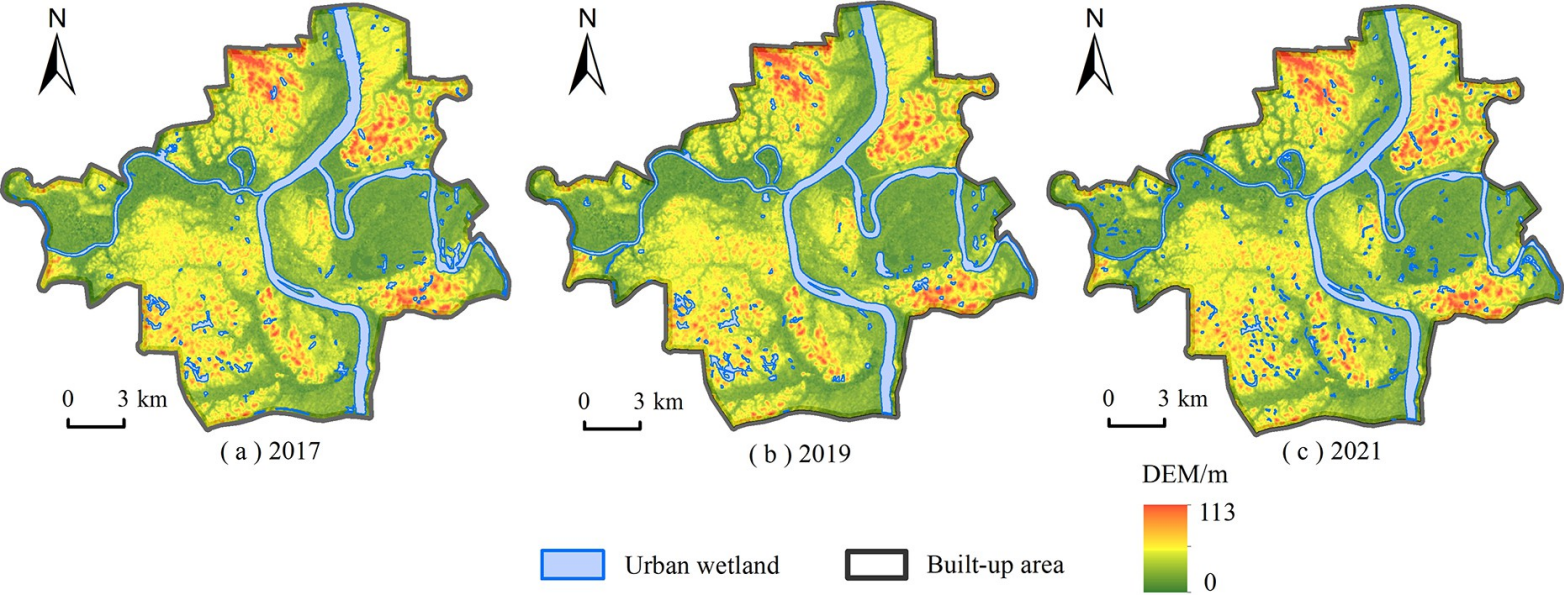
Distribution of wetlands in the study area. (a) Results of urban wetland extraction in 2017; (b) Wetland extraction in 2019; (c) Wetland extraction in 2021.

In terms of the work flowchart (Fig 2), we utilized the AI Earth Platform to extract the urban wetlands in the built-up areas of the main urban areas of Hengyang City. Through the related results (Table 2), there is an obvious and stable decreasing trend of the total area of urban wetlands in the built-up areas. Simultaneously, an apparent and more fragmented trend of the spatial distribution of urban wetlands in the built-up areas can also be observed (Fig 3). In each administration district of the main urban areas, the area of urban wetlands is decreasing year by year.

**Table 2:** The extraction results of the study area in 2017, 2019 and 2021.

Year	Wetland area in built-up area(ha)
**2017**	3246.08
**2019**	3221.62
**2021**	2973.42
**average**	3147.04

### WESV results under different correction factors

This article estimates WESV using three methods, namely uncorrected WESV, WESV corrected based on precipitation, and WESV corrected based on water production.

#### Uncorrected WESV results

According to the statistical yearbook in 2017, 2019, and 2021, which was delivered by the Hengyang City Government, this work first calculated the total area of grain growing, gross grain production, and the related grain purchase prices. Then, this work accordingly determined the average value of grain per ha (or per unit area) in the main urban areas of Hengyang City. In 2017, the value was 14733.74 yuan/ha; in 2019, the value increased to 13968.42 yuan/ha; in 2021, the value kept consecutively growing and reached 11548.96 yuan/ha. Calculate the uncorrected WESV results (Table 3) in the built-up areas of the main urban areas of Hengyang City in 2017, 2019, and 2021 based on Formulas (6), (9) and (10).

**Table 3:** The uncorrected WESV results of the study area from 2017 to 2021 (100 million yuan).

Ecosystem classification		2017		2019	2021
**Regulating services**	Air quality regulation		0.37	0.34	0.26
	Climate regulation		1.10	1.01	0.79
	Water regulation		48.90	45.11	35.11
	Waste treatment		2.65	2.45	1.91
**Provisioning services**	Food supply		0.38	0.35	0.27
	Raw material supply		0.11	0.10	0.08
	Water supply		3.96	3.66	2.85
**Supporting services**	Soil conservation		0.44	0.41	0.32
	Biodiversity		1.22	1.13	0.88
	Nutrient cycling		0.03	0.03	0.02
**Cultural services**	Cultural & amenity services		0.90	0.83	0.65
	Total		60.08	55.42	43.13

Through Table 3, the total results of WESV in the built-up areas of the main urban areas of Hengyang City decreased sharply, from 60.08 in 2017 to 43.13 hundred million in 2021. Then, the most important ecosystem services function of urban wetlands in the study area is hydrological regulations. The share of hydrological regulations in total WESV was about 80.42% in 2017, less than 81.40% in 2019, and more than 81.40% in 2021. Obviously, hydrological regulations increase slowly although the total WESV in the study area continuously decreases. Besides, the water supply is the second most important ecosystem services function of urban wetlands in the study area. Distinguished from the hydrological regulations, water supply shows the same variation trend as total WESV. This suggests that the water supply constantly reduces from 2017 to 2021. So do the other functions such as air quality regulations, climate regulations, waste treatment, etc.

#### WESV results based on precipitation correction

According to Xie G.D. et al. [15], precipitations can widely reflect the dynamic change features from the spatial and temporal dimensions. Combined with the basic equivalent table of ecosystem service value, the dynamic value equivalent table of urban wetland ecosystem service based on precipitation factor correction was constructed, and the spatial and temporal adjustment factors of precipitation were obtained according to Formula (7). Hence, this work establishes the dynamic value equivalent table for WESV in the study area (Table 4).

**Table 4:** Precipitation and correction factors for WESV from 2017 to 2021.

Year	Precipitation(mm)	Spatial and temporal regulator factor of precipitation
**2017**	1412.05	0.77
**2019**	1576.29	0.86
**2021**	2526.60	1.37
**Annual average**	1838.31	1.00

From 2017 to 2021, the precipitations of Hengyang City increased fast. Especially, the precipitation in 2021 is almost twice that in 2017. This strongly hints that the climate changes are distinct and have a big impact on the WESV results of Hengyang City. According to Table 4, although the gap in the value of the precipitation regulation factor between 2017 and 2019 is not obvious, there is a significant difference between 2021 and 2017, 2019. Both of 2017 and 2019, the values are less than 1. However, the value is more than 1.4 in 2021. Combined with Table 4, Formula (9) and Formula (10), the WESV results based on precipitation correction in the study area were obtained (Table 5).

**Table 5:** Total WESV based on precipitation correction of the study area from 2017 to 2021 (100 million yuan).

Ecosystem classification		2017	2019	2021
**Regulating services**	Air quality regulation	0.37	0.34	0.26
	Climate regulation	1.10	1.01	0.79
	Water regulation	37.65	38.68	48.25
	Waste treatment	2.65	2.45	1.91
**Provisioning services**	Food supply	0.38	0.35	0.27
	Raw material supply	0.11	0.10	0.08
	Water supply	3.05	3.14	3.91
**Supporting services**	Soil conservation	0.44	0.41	0.32
	Biodiversity	1.22	1.13	0.88
	Nutrient cycling	0.03	0.03	0.02
**Cultural services**	Cultural & amenity services	0.90	0.83	0.65
	Total	47.92	48.47	57.35

From Table 5, it is clear that the water regulation, water supply, and total WESV results are increasing. At the same time, although the other ecosystem service functions of urban wetlands in the study area are all decreasing, they still have great differences. For example, both values of nutrient cycling in 2017 and 2019 are the same; however, in 2021, nutrient cycling still decreases. Through the comparison of total WESV with 2017, 2019, and 2021, the increasing speed is apparently distinct. Compared to 2017, there is only an increment of 0.55 × 10^8^ yuan in 2019. However, there comes a sharp increment of 8.88×10^8^ yuan in 2021 compared to 2019. In addition, through Table 5, the water regulation factor is the most significant ecosystem service function of urban wetlands in the study area because it contributes the biggest part; water supply is the second most important ecosystem service function; and waste treatment occupies the third place. Hence, it can be observed that the hydrological regulation function remains the most crucial service function for the WESV assessment, constituting approximately 82% of the total.

#### WESV results based on water yield correction

**Fig 4:**
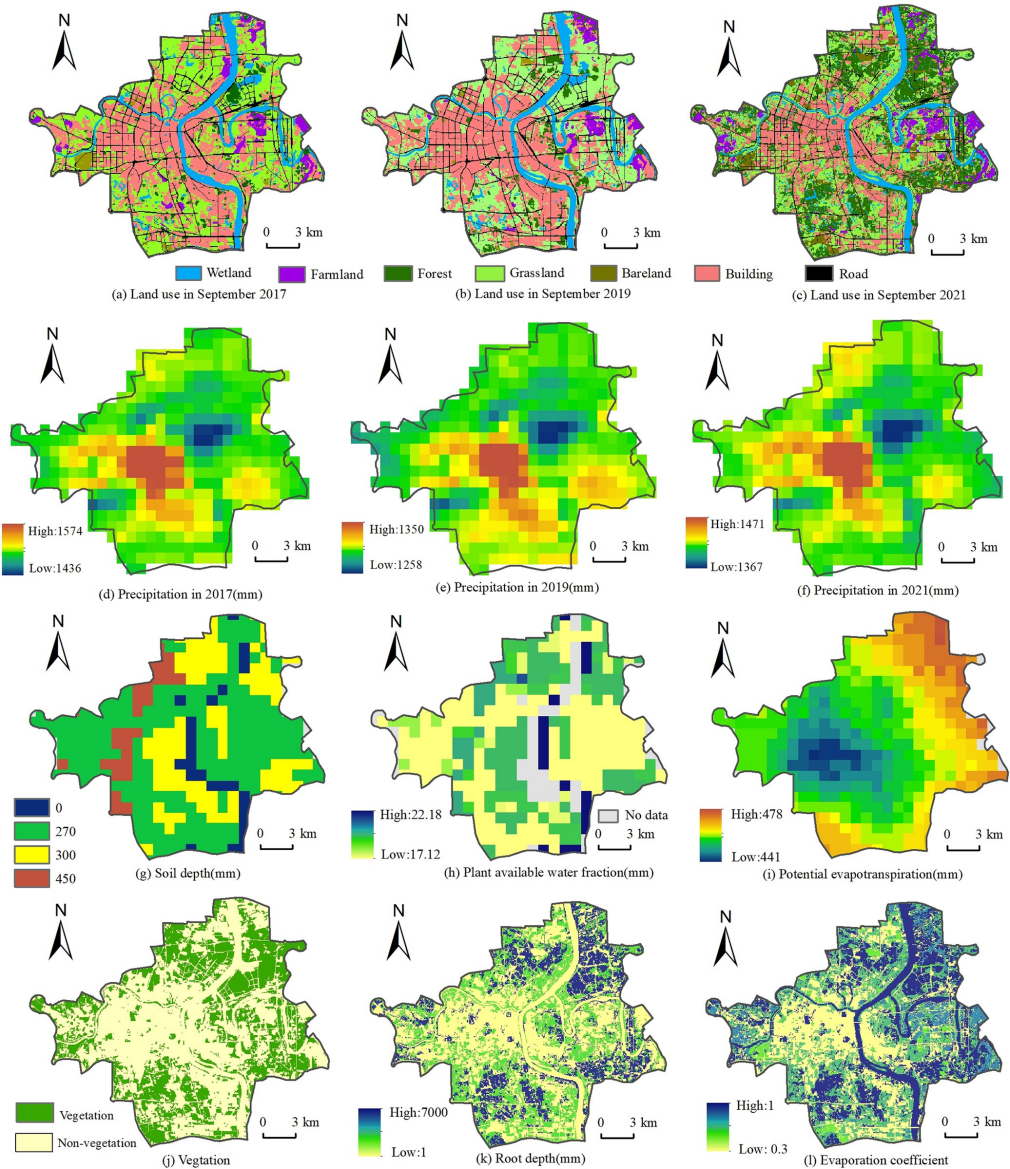
The factors of water yield model. (a)(b)(c) Results of land use extraction in September 2017, 2019 and 2021 respectively; (d)(e)(f) The distribution of precipitation in 2017, 2019 and 2021 respectively; (g) Soil depth is the distance from the surface to the underground rock or mineral layer, (h) Plant available water fraction, the difference between field capacity and wilting point; (i) Potential evapotranspiration is the amount of heat that a substance needs to absorb as it transitions from a liquid state to a gaseous state; (j) The vegetation type is assigned to 1, and other land uses are 0; (k) The root depth of soil refers to the maximum depth that plant roots can extend in the soil; (l) The evaporation coefficient is based on the reference evaporation given by the physiological characteristics of alfalfa plants.

**Fig 5:**
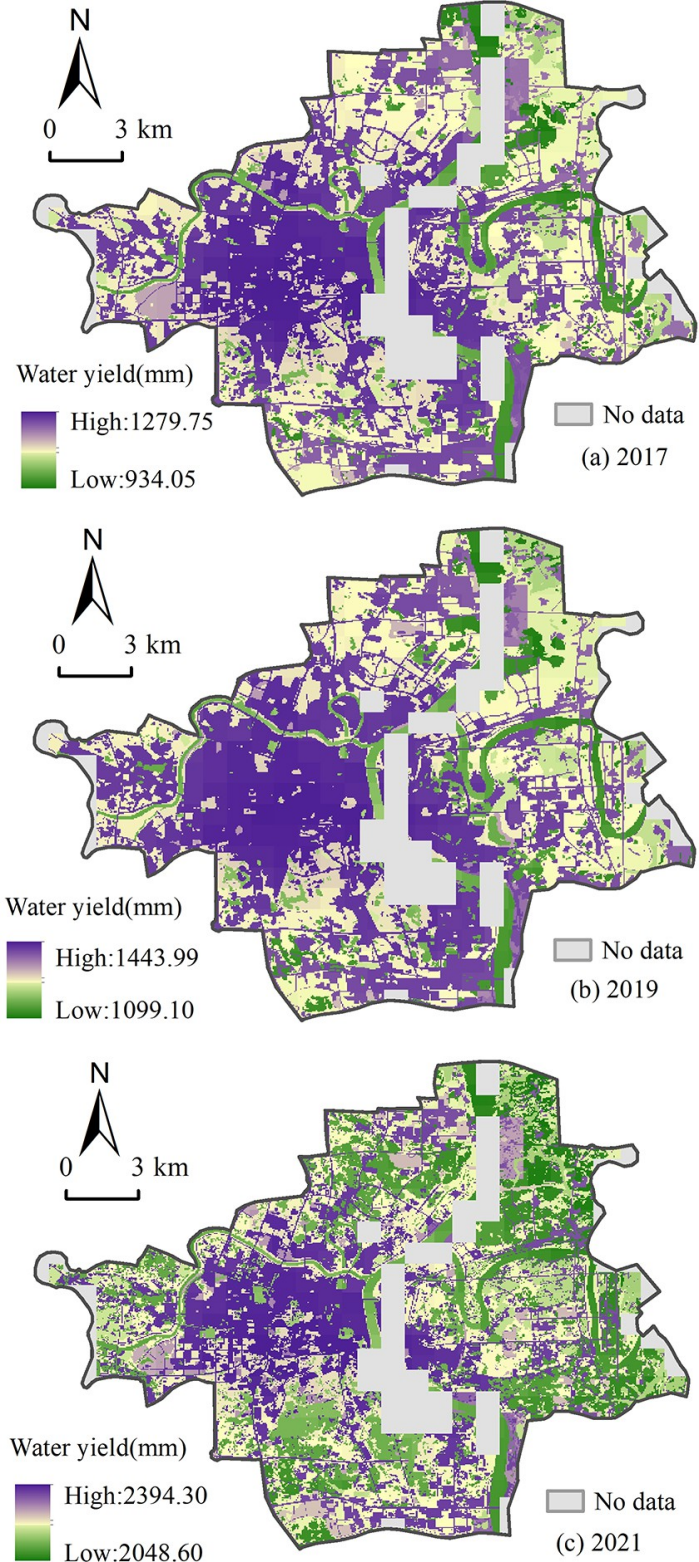
Annual water production of the study area in 2017, 2019 and 2021.

There must be a deviation between the WESV assessment results of the study area and the related true values because the variations of different annual precipitations show great differences. Note that, compared to the precipitations, the water yield factor can reflect more environmental features and more stable. In order to reduce the errors of WESV assessment results, hence, this work uses water yield factor per unit area to replace the precipitations per unit area. Through running the water yield module of InVEST [44], this work determines the water yield factor (Fig 5) via combining the related factors (Fig 4), including precipitations, soil layer depth, land uses types, evapotranspiration, and PAWC.

Through Fig 4(A)–4(C), the building land in the study area mainly occupy the areas from Leishui south to Xiangjiang river. Regarding precipitation, based on Fig 4(D)–4(F), it can be observed that the areas of concentration and high values are found in the southern and central regions of the study area, while the remaining areas are characterized by low values. Besides, Fig 4 also show that the spatial distribution patterns of average annual potential evapotranspiration like a circle, the closer to the center, the lower to the value. It is worthy to note that the differences of average annual potential evapotranspiration between the high value and low value are not prominent, however, only 37 mm.

Fig 5 depicts the spatial distributions features and temporal changes characteristics of the water yield factors of the study area from 2017 to 2021. At first, although the values of water production in the study area in each investigation year are distinct, the related differences between the high value and low value are very closing, 345.7 mm in 2017, 344.89 mm in 2019, and 345.7 mm in 2021, respectively. This suggests that the building land in the study area dramatically impacts regional water production. For example, in urban areas, the roads, streets, alleys, and most of the grounds are usually covered by waterproof surfaces, this can tremendously change and even cut off the natural permeation processes of the rainwater. Note that, in the urban area, the rainwater can’t permeate into the ground of the built-up areas compared to the natural surfaces. This leads to the ground runoff in the built-up areas increasing so that people have to build more sewers, pipelines, ponds, and even man-made lakes in the cities. At the same time, this also affects the natural evapotranspiration processes in urban areas. In Fig 5, in the built-up areas of the study area, the building land is more concentrated, and the natural evapotranspiration is less; on the contrary, the corresponding water production is far more. Secondly, according to the distributions of built-up areas in the study area, a spatial correlation of distribution between the built-up areas (Fig 1) and water production of the study area in each investigation year (Fig 5), both of them are intensively distributed and highly overlapped, can be confirmed. This directly affirms that the built-up areas of urban regions can greatly affect and even change regional water production.

In terms of the above and simultaneously linking the water yield factor (Formula 8), the water yield factor of the study area is determined for each investigation year (Table 6). According to the results, from 2017 to 2021, the water yield factor shows a stable increasing trend. Especially, compared to 2017, the water yield factor in 2021 almost double. In addition, compared to the precipitations, the water production reduces sharply due to the influencing factors of evapotranspiration, PAWC, land use, and soil layer depth.

**Table 6:** Water yield and correction factors for WESV from 2017 to 2021.

Year	Water Yield(mm)	Spatial and temporal regulator factor of Water Yield
**2017**	1139.34	0.74
**2019**	1309.73	0.85
**2021**	2196.96	1.42
**Annual average**	1548.68	1.00

Based on the water yield factor in each investigation year, this work calculates the WESV per unit area of urban wetlands of the study area by combining Formula 9 and Formula 10 (Table 7). Through these results, the total values of WESV per unit area of the study area keep consecutively increasing. And the increments are also fast increasing. Compared to 2017, the increments in 2019 are 100, 000 yuan/ha. However, in 2021, the increments reached 460,000 yuan/ha compared to 2019. Besides, Table 8 suggests that both water regulations and water supplies are increasing while the others keep continuously decreasing.

**Table 7:** The unit area of WESV based on water yield correction of the study area from 2017 to 2021 (10,000 yuan/ha).

Ecosystem classification		2017	2019	2021
**Regulating services**	Air quality regulation	1.13	1.08	0.89
	Climate regulation	3.37	3.21	2.63
	Water regulation	110.57	121.05	166.79
	Waste treatment	8.16	7.77	6.38
**Provisioning services**	Food supply	1.18	1.12	0.92
	Raw material supply	0.34	0.32	0.26
	Water supply	8.97	9.82	13.52
**Supporting services**	Soil conservation	1.37	1.30	1.07
	Biodiversity	3.75	3.57	2.93
	Nutrient cycling	0.10	0.10	0.08
**Cultural services**	Cultural & amenity services	2.78	2.65	2.17
	Total	141.70	151.98	197.66

**Table 8:** Total WESV based on water yield correction of the study area from 2017 to 2021 (100 million yuan).

Ecosystem classification		2017	2019	2021
**Regulating services**	Air quality regulation	0.37	0.34	0.26
	Climate regulation	1.09	1.01	0.78
	Water regulation	35.89	38.24	49.59
	Waste treatment	2.65	2.45	1.90
**Provisioning services**	Food supply	0.38	0.35	0.27
	Raw material supply	0.11	0.10	0.08
	Water supply	2.91	3.10	4.02
**Supporting services**	Soil conservation	0.44	0.41	0.32
	Biodiversity	1.22	1.13	0.87
	Nutrient cycling	0.03	0.03	0.02
**Cultural services**	Cultural & amenity services	0.90	0.84	0.65
	Total	46.00	48.01	58.77

At the same time, this work also uses the water yield factor to correct the WESV assessment results of the study area (Table 8). The corrected WESV assessment results with the water yield factor increased 2×10^8^ yuan from 2017 to 2019, and then continuously increased 10.76×10^8^ yuan from 2019 to 2021. This clearly hints that the WESV of the study area shows an active role in improving the environmental qualities. For example, according to our results, the hydrological regulations, water resources supplies, and environmental purification have also apparently improved from 2017 to 2021. From the perspective of the types of ecosystem services, there are many great active changes in some ecosystem services, such as hydrological regulations, water resources supplies, biodiversity, aesthetic landscapes, etc.

#### The comparisons among the uncorrected results, corrected results with precipitations and corrected results with water yield factor

**Fig 6:**
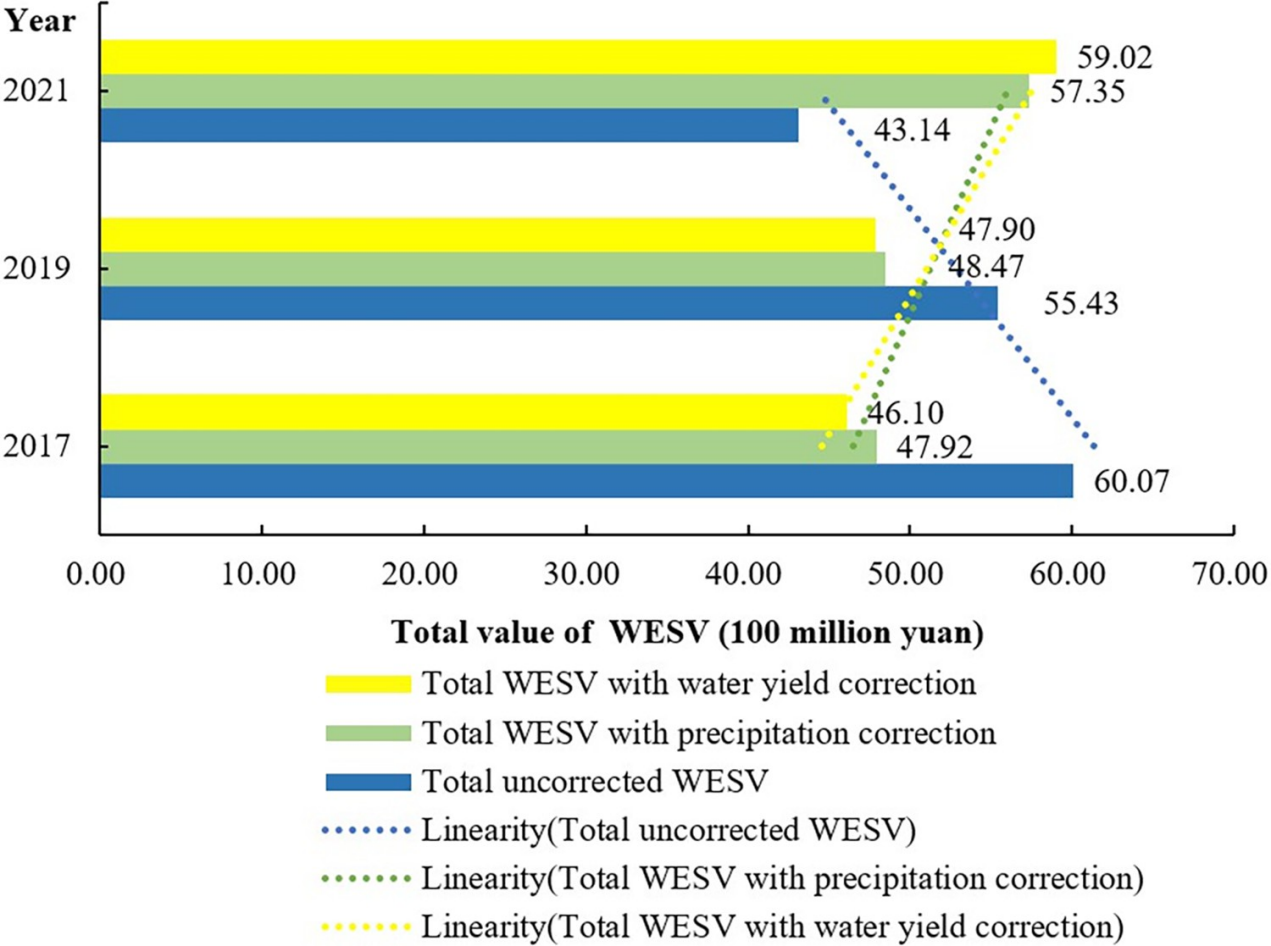
Comparison of total WESV with different correction factors from 2017 to 2021.

In order to make clear the differences between the uncorrected and corrected results with various methods on the WESV assessment of the study area, this work carries out the comparisons among the uncorrected results, corrected results with the precipitations, and corrected results with the water yield factor. Firstly, according to the comparisons, the uncorrected results show a continuous decreasing feature (Fig 6). From 2017 to 2019, there is a reduction of 4.64×10^8^ yuan; and then, from 2019 to 2021, there is also a reduction of 12.29×10^8^ yuan. However, at the same time, all the corrected results show an opposite feature compared to the uncorrected results. In other words, they keep increasing over the whole investigation time. It is noteworthy to stress that there are significant differences existing in the two correction methods through this work. On the whole, according to the comparisons, the water yield factor can reflect more environmental features than the precipitations when determining the WESV. Because the water yield factor comprehensively reflects the environmental features at the regional scale, such as precipitations, soil layers, land uses types, and PWAC, etc. This thus means that the water yield factor can be more helpful to reduce the errors than the precipitations when correcting the WESV assessments. From this point, this work tries to employ the water yield regulation factor (including water resources supplies, hydrological regulations) to correct the value equivalent factors. Of course, this also hints that we can seek more appropriate correction indices can be found to support the accurate WESV assessment and provide good advice on the urban ecological planning in the future.

### Analysis of the influencing factors

**Fig 7:**
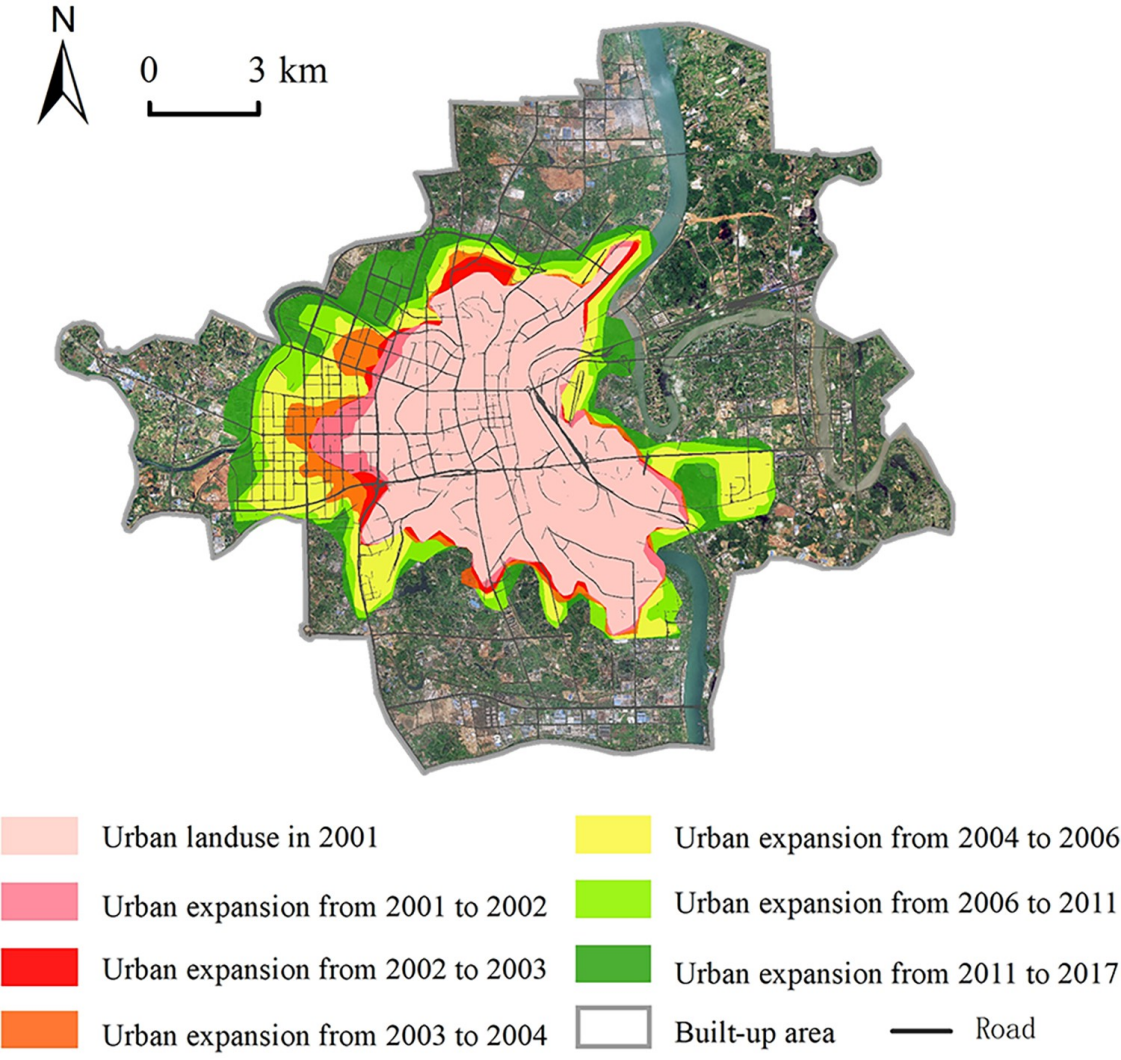
Maps of the expansion process of the study area from 2001 to 2017 (According to literature [45] and dataset [47]). Republished from Journal of Global Change Data & Discovery under a CC BY license, with permission from Chuang Liu, original copyright 2020.

This paper used the urban expansion dataset of the main urban area of Hengyang City from 2001 to 2017 to show the urbanization expansion process of the central urban area of Hengyang City in recent years (Fig 7) [45]. It can be seen that the urban expansions of the study area were mainly concentrated along the main roads in the city and on both sides of the rivers. During the continuous developments and expansions of urban areas, more and more wetlands or small wetlands [46], which had been located in the suburban areas during the past decades, have been heavily involved in the construction since 2001. Because these wetlands are usually located on the two sides of the rivers are flat and low.

On the other hand, more and more local residents and outside tourists intend to explore the beautiful landscapes and scenes of urban wetlands near the city with increasing incomes and leisure time than ever before. For example, during the past decade, Hengyang City had built and opened tens of theme parks, such as Maya Beach Water Park was opened in July 2022. However, the flourishing tourism on urban wetlands inevitably brings a couple of issues, such as rubbish, construction wastes, soil erosion, and so on. Note that, building water theme parks or urban wetland parks may affect the water cycle in the built-up areas of the study area. Because the water theme parks or urban wetland parks usually need to drain or store the ground water in the built-up areas when they are in running. Let us take Nanhu Park as an example. Nanhu Park is the biggest water theme park in the main urban area of Hengyang City and its water area is about 33.3 ha. Since its opening, Nanhu Park has attracted about 15 million visitors each year. However, it must be acknowledged that, despite the significant economic benefits brought in annually by Nanhu Park, the management of tons of wastes and rubbish generated by numerous visitors falls under the responsibility of the city sanitation departments. This suggests that tourism developments on the urban wetlands can affect their stability and ecosystem services functions. Finally, the territorial spatial planning and city development planning can also bring important affections on the urban wetlands in the built-up areas of the study area. For example, according to the 14th Five-Year-Plan of Hengyang City, Zhuhui District launched the construction of Linghu Water Park in end of 2022.

## Section 5: Conclusions

To a large extent, the urban wetlands can be seen as ecological infrastructures and excellent assets of social development to support regional sustainable developments. It thus is of crucial implications to improve and explore the appropriate assessment methods or models to catch the natural features of WESV. This can help us precisely assess the WESV and further provide enough knowledge to understand their ecosystem services features. The research findings are as follows: the unadjusted Wetland Ecosystem Service Value (WESV) in the built-up area of the central urban area of Hengyang City decreased from 60.07 billion yuan in 2017 to 43.14 billion yuan in 2021. However, the WESV based on precipitation adjustment increased from 47.92 billion yuan in 2017 to 57.35 billion yuan in 2021, and the WESV based on water yield adjustment increased from 46.1 billion yuan in 2017 to 59.02 billion yuan in 2021. Through the case study, this work confirms an effective approach to improve the accuracy of WESV assessment via using the water yield factor. From this work, the following aspects are of important roles in future research.

The research highlights the effectiveness of using the water yield factor for accurate WESV assessments. Value equivalent factors improve precision by aligning results with the reality of ecosystem service functions, including precipitations, water production, and water supplies. The water yield factor outperforms the single precipitation factor, capturing temporal and spatial dimensions of urban wetlands’ ecosystem services, considering factors such as soil, land use, and PWAC. In contrast, relying solely on annual average precipitations challenges accurately depicting WESV. Significant differences emerge between precipitation regulation and water yield factor corrections, impacting climate regulation, hydrological regulation, water supplies, biodiversity, soil conservation, and aesthetic landscapes. This insight inspires exploration of influencing factors in future WESV assessments.

It is clear that this work will be of great meaning to further capture the ecosystem services values and even make the pertinent decisions to protect and promote sustain-able developments for the urban wetlands. However, it is also outstanding to point out that although our research findings support using the water yield factor can obtain more significant results when correcting WESV assessment, there is still much room to continuously improve the existing methods and the related assessment results of this work in our next researches.
